# Reflections on 50 years of immunisation programmes in the WHO African region: an impetus to build on the progress and address the unfinished immunisation business

**DOI:** 10.1136/bmjgh-2024-017982

**Published:** 2025-05-21

**Authors:** Amos Petu, Balcha Masresha, Charles S Wiysonge, Jason Mwenda, Kwasi Nyarko, Ado Bwaka, Sarah Wanyoike, Franck Mboussou, Benido Impouma, Abdulmumini Usman, Olushayo Oluseun Olu, Alex Ntale Gasasira, Joseph Waogodo Cabore, Matshidiso R Moeti

**Affiliations:** 1 World Health Organization Regional Office for Africa, Brazzaville, Congo; 2 Vaccine Preventable Diseases Unit, World Health Organization Regional Office for Africa, Brazzaville, Republic of Congo, Congo; 3 Department of Global Health, Stellenbosch University, Stellenbosch, South Africa; 4 World Health Organization Intercountry Support Team for West Africa, Ouagadougou, Burkina Faso; 5 World Health Organization Intercountry Support Team for East and Southern Africa, Harare, Zimbabwe; 6 Vaccine Preventable Diseases Unit, World Health Organization Intercountry Support Team for Central Africa, Libreville, Gabon; 7 Office of the Director of Programme Management, World Health Organization Regional Office for Africa, Brazzaville, Brazzaville, Congo; 8 Office of the Regional Director, World Health Organization Regional Office for Africa, Brazzaville, Congo

**Keywords:** Immunisation, Africa South of the Sahara, Public Health, Child health

## Abstract

Immunisation is crucial to achieving the Sustainable Development Goals for maternal and child mortality reduction. As Africa marks the 50th anniversary of implementing immunisation programmes, it is imperative to review progress, address challenges and strategise for the future. Using available programme data, this article examines the progress made in achieving the immunisation milestones in the region, describes the success factors and lessons learnt and makes recommendations on how to immunise every African child in the coming years. The article concludes that despite significant improvements in childhood immunisation coverage, the region still falls short of global targets, with disparities across countries. Contributing factors include, among others, weak health systems, rapid population growth without corresponding increases in service delivery, vaccine hesitancy, inadequate sustainable financing and disruptions caused by the COVID-19 pandemic. Moving forward, efforts to attain the global immunisation coverage milestones should focus on building on the past progress, addressing the COVID-19 setbacks, leveraging new technologies and securing sustainable immunisation funding. This can be achieved by accelerating the implementation of the Immunization Agenda 2030 and the Addis Ababa Declaration on Immunization commitments. The support of all stakeholders including political leaders, public health professionals, the vaccine industry, regional organisations, academia, donors and healthcare workers is essential for this noble endeavour.

**Summary box**

- Vaccines and immunisation are among the most impactful and cost-effective public health interventions, with initiatives such as the Expanded Programme on Immunisation and the Global Polio Eradication Iinitiative significantly improving immunisation delivery and coverage in Africa since 1974.
- Despite progress, Africa still falls short of global childhood immunisation targets, with persistent country-level disparities driven by weak health systems, rapid population growth, vaccine hesitancy, inadequate financing, and COVID-19 disruptions.
- This study underscores the need for accelerating the implementation of the 2030 Immunisation Agenda and the Addis Ababa Declaration on Immunisation commitments, addressing the COVID-19 setbacks, and securing sustainable domestic immunisation funding to enhance immunisation coverage across the region. Additionally, implementation research should be scaled up to identify and fill service delivery gaps and promote the adoption of innovative technologies.

## Introduction

 Since their discovery in 1796, vaccines have evolved into one of the most impactful and cost-effective public health interventions globally. ^1 2^They have revolutionised global public health practice and contributed to several global public health milestones such as the global eradication of the smallpox virus,^3^ containment of the COVID-19 pandemic,^4^ control of Ebola virus disease (EVD) outbreaks in Africa^5^ and the achievement of the Millennium Development Goals in several countries.^6^ Additionally, they remain the most potent tool in the imminent global eradication of polio^7^ and in the prevention and control of at least 25 diseases.^8^

**Figure 1:**
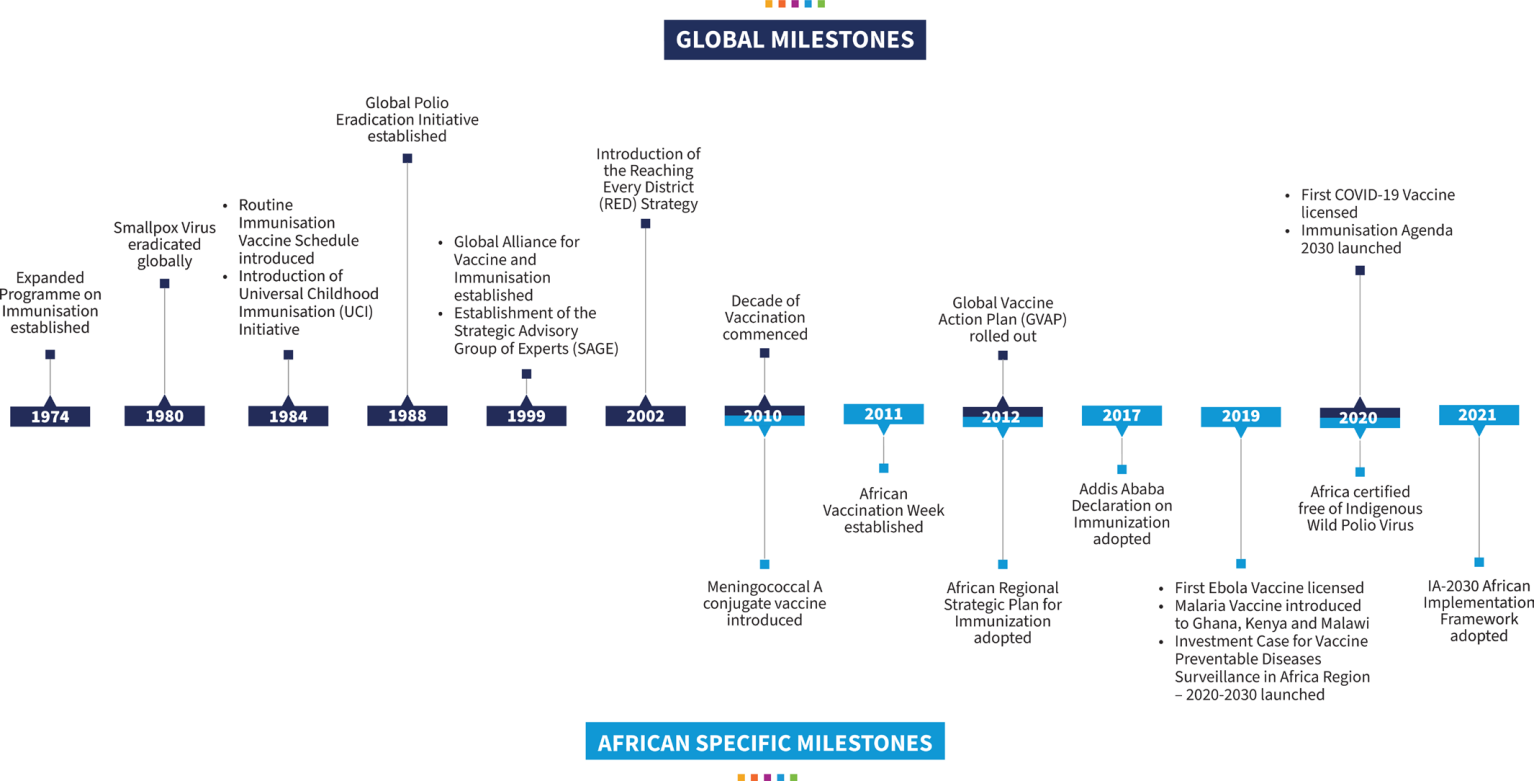
Global and African immunisation milestones: 1974–2024.

The global Expanded Programme on Immunization (EPI), established in 1974, introduced a standardised childhood immunisation schedule against six childhood killer diseases in 1984 (figure 1).^9^ This schedule has expanded from six to over 13 antigens (Bacillus Calmette-Guerin (BCG), Diphtheria, Pertussis and Tetanus (DPT), *Haemophilus influenzae* type B, hepatitis B, polio, measles, rubella, pneumococcal disease, rotavirus, human papillomavirus (HPV), COVID-19 (for adults)), adopting a life-course approach to vaccination.^10^ The Global Polio Eradication Initiative (GPEI), launched in 1988, has been instrumental to the massive increase in the numbers and development of the immunisation workforce, scaling up of vaccine preventable disease (VPD) control activities and strengthening national immunisation programmes in general.^11^,^13^ In 1999, a global coalition comprising United Nations agencies, the World Bank, donors, international non-governmental organisations and countries established the Global Alliance for Vaccines and Immunisation (Gavi, the Vaccine Alliance), to ensure that immunisation reaches every child, particularly those living in the least developed countries (figure 1).^14^

All the 47 member States of the WHO Africa Region (WHO/AFR) have been implementing the EPI programme from inception and have launched several continental initiatives aimed at boosting childhood immunisation such as the Reaching Every District (RED) strategy in 2002, the yearly African vaccination week in 2011 and the Addis Ababa Declaration on Immunization (ADI) in 2017 (figure 1). The landmark ADI, among others, committed African governments to accelerate actions towards sustainable financing of immunisation, increased efficiency of immunisation delivery systems, maintaining high quality VPD surveillance and self-reliance in access to vaccines.^15^

As the 50th anniversary of EPI is being marked, Africa must review progress, address the pervasive challenges and strategise for the future of immunisation on the continent for a few reasons. First, immunisation is key to the achievement of the Sustainable Development Goals (SDGs), including Universal Health Coverage (UHC); thus, there is the need to define strategies for strengthening and scaling up service provision towards attaining equity in universal coverage in the run-up to 2030.^16^ Second, immunisation will continue to play important roles in the prevention and control of new and re-emerging pathogens in the region; hence, there is the need for dialogue on how the continent could ensure self-reliance in vaccines demand and supply. Third, the wide disparities in vaccine equity in the region require unpacking with a view to designing appropriate strategies for closing the gaps. Fourth and importantly, the lessons learnt and best practices over the past five decades would guide future immunisation efforts and policy decisions.

While several authors have conducted similar reviews, none have covered the full 50-year EPI implementation period. Okesanya *et al* examined immunisation challenges in Africa from an academic perspective,^17^ while Mihigo *et al* reviewed the situation as of 2015 and 2017.^18 19^ Furthermore, these studies did not comprehensively address the issue of sustainable immunisation financing. The current analysis aims to review the available evidence and update previous research on immunisation over the last 50 years of EPI implementation in the 47 countries of WHO/AFR. It highlights the success factors, lessons learnt and offers practical and comprehensive policy, strategic and operational recommendations for ensuring that every African child receives immunisation in the coming years.

## Progress towards achievement of the immunisation milestones in the African region

### Vaccine access and immunisation coverage

**Figure 2:**
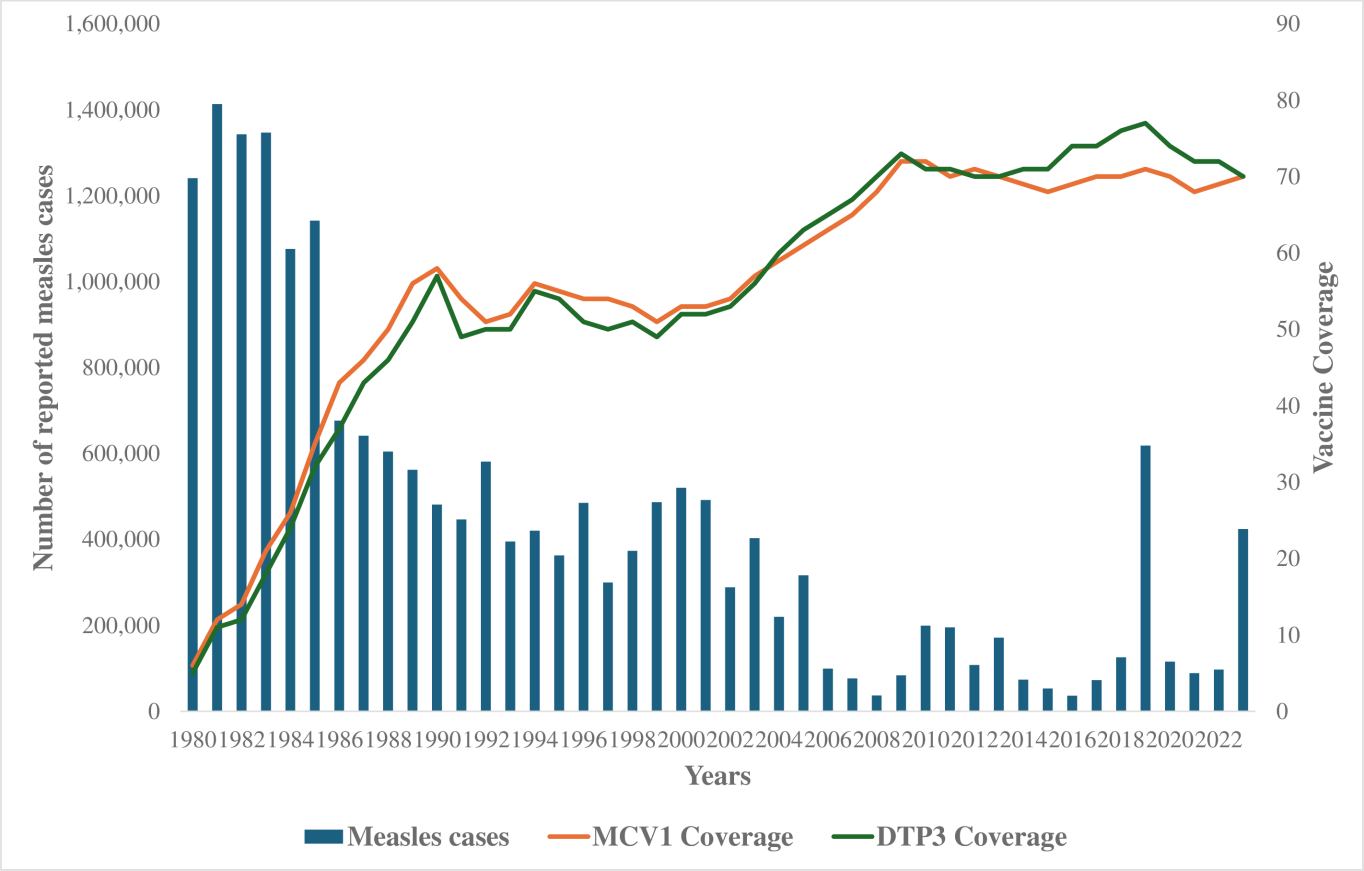
Coverage of the first dose of the Measles Containing Vaccine (MCV1) and third dose of the Diphtheria, Pertusis and Tetanus Vaccine (DPT3) (WUENIC) and number of officially reported measles cases in the WHO African region—1980 to 2023.

The establishment of the global EPI led to an increase in the vaccines antigens on offer and increased the third dose of DPT (DPT3) and first dose of Measles Containing Vaccine (MCV1) coverage rates from less than 10% in 1980 to about 57% in 1990 and then to more than 70% in 2022 (figure 2). There has also been an expansion of the portfolio of vaccines to include new and more expensive vaccines such as the HPV and malaria vaccines. However, even though annually more children were being reached with these antigens, the regional coverage rates with DPT3 and MCV1 have stagnated since 2010 (figure 2). Africa faces a significant challenge in reducing the high number of unvaccinated and undervaccinated children. In 2021 alone, 12.7 million children were underimmunised, including 8.7 million who did not receive a single vaccine dose, commonly referred to as zero-dose children.^20^ Alarmingly, half of the 20 countries with the highest number of zero-dose children globally are in Africa, with Nigeria and Ethiopia carrying the heaviest burden—over 2.2 million and 1.1 million zero-dose children, respectively.^20^

The persistent stagnation in regional MCV1 and DPT3 immunisation coverage rates at 70% from 2010 to date and the increase in zero-dose and underimmunised children could be attributed to a few factors. First, anecdotal evidence shows that the rapid population growth in the region is not met by a commensurate increase in immunisation service delivery. Second, serious infrastructure constraints and/or high levels of insecurity due to conflicts and civil wars and uncontrolled urbanisation have reduced access to immunisation services in some countries of the region.^21^ Third, sociocultural factors continue to result in various degrees of vaccine hesitancy.^22^ For instance, several communities across many African countries do not take their children for any vaccination due to sociocultural reasons.^23^ Fourth, the GPEI ramp down, which started in 2020, reduced resources available to immunisation programmes, thus impacting negatively on service delivery, leading to suboptimal coverage in several countries.^24^ Fifth, the high illiteracy levels and inadequate awareness about the benefits of immunisation, particularly among African caregivers, remain a challenge to seeking immunisation services.^25 26^ Sixth, the COVID-19 pandemic and recurrent EVD epidemics have, at different times, resulted in the general disruption of health and immunisation services delivery.^27^,^29^ Seventh, competing public health problems such as emerging non-communicable and communicable diseases, including cholera, EVD and COVID-19, that equally require vaccination continue to put pressure on the available resource envelope, leading to possible reduction in the availability of routine immunisation resources.

**Figure 3:**
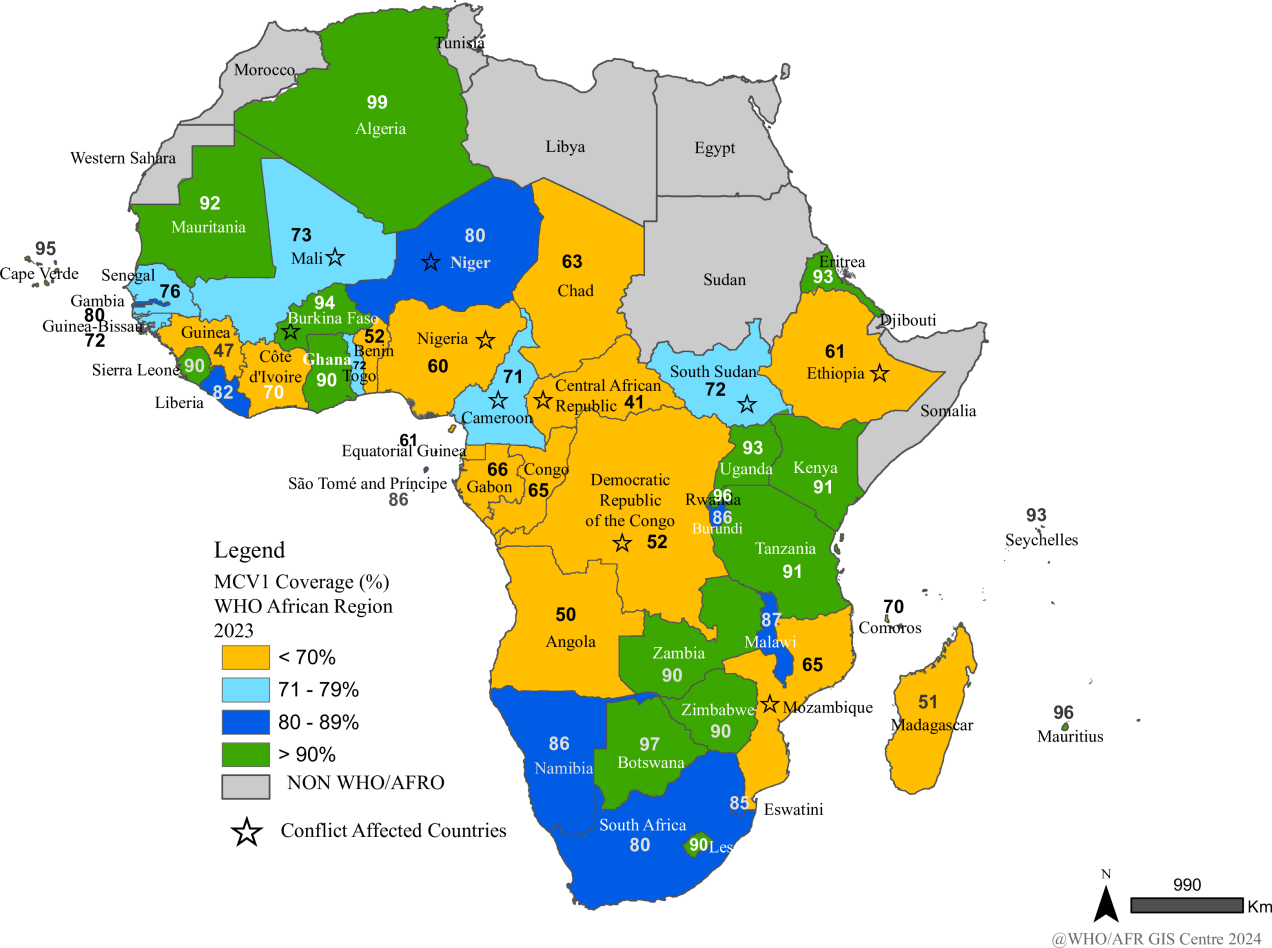
MCV1 coverage in the WHO African region—WUENIC, 2023. AFRO, African regional office.

Furthermore, the 2023 estimates show intercountry disparities in coverage rates (figure 3). For instance, almost all conflict-affected and fragile countries had less than 70% coverage with the Central African Republic (CAR) (41%), Democratic Republic of Congo (DRC) (52%), Ethiopia (61%), Mozambique (65%) and Nigeria (60%) recording the lowest MCV1 coverage rates in the region (figure 3). While as of 2023, Algeria (99%), Botswana (97%), Cabo Verde (95%), Mauritius (96%) and Rwanda (96%) attained over 95% MCV1 coverage rate which is recommended to meet the measles elimination strategy targets (figure 3). The fact that Nigeria, Ethiopia, Angola and DRC have low coverage rates has a huge impact on the regional weighted average as these four countries make up more than 40% of the target population in the Region. The observed intercountry disparities in immunisation coverage are products of demographic complexities such as the high birth rate resulting in a large young population, changing denominator of immunisation coverage calculation and fluid population movements which existing immunisation services cannot address. Other reasons include the differing status of health systems service delivery capacity and domestic funding of routine immunisation.^30^ These are further compounded by the political and security situations which disrupt the health system’s integrity in countries experiencing armed conflicts.

These coverage gaps have given rise to outbreaks of VPDs across the continent at different times. In the last two decades, countries with suboptimal measles vaccination coverage rates including CAR,^31^ DRC,^32^ Ethiopia,^33 34^ Madagascar,^35^ South Sudan,^36^ Chad, Nigeria^37^ and Niger^38^ have reported repeated measles outbreaks and a tendency of increasing measles incidence, while epidemics of diphtheria have been reported in Cameroon, Guinea, Mauritania, Nigeria, Niger and South Africa.^39 40^

### Immunisation financing

**Figure 4:**
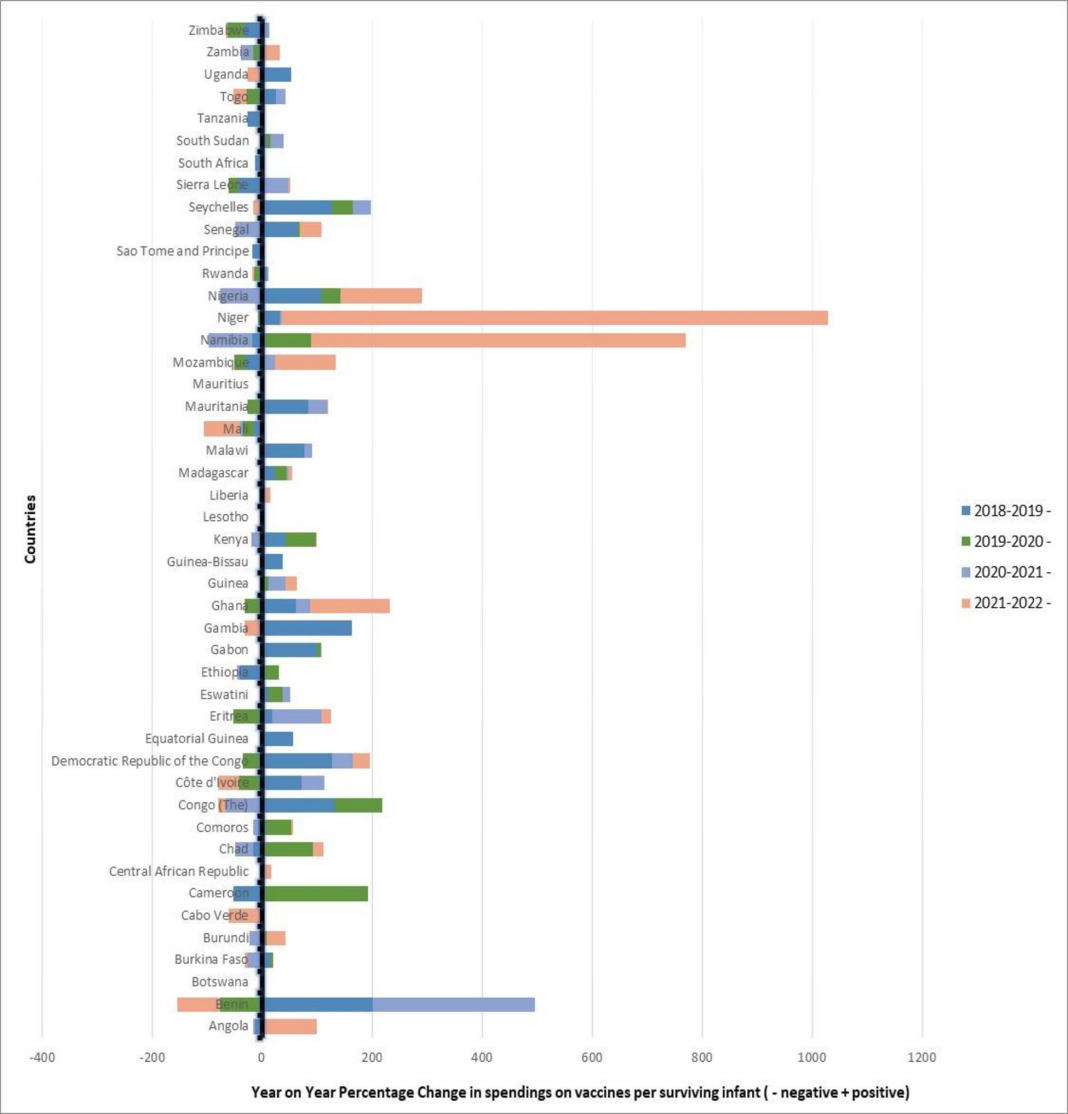
Year-to-year changes in total spending on vaccines from all sources per surviving infant in the WHO African region—2018 to 2022.

Over the past 50 years of immunisation programme implementation, there has been a global increase in the financing of immunisation services. Available data on the African Region indicate that although absolute funding on immunisation has increased, the trend in total spending on vaccines from all sources (government and others) per surviving infant has been fluctuating (figure 4).^41^ In 9 (Benin, Burkina Faso, Congo, Cote d’Ivoire, Mali, Rwanda, Seychelles, Togo and Zimbabwe) out of 34 countries, in 2022, there was a reduction in total spending on vaccines used in routine immunisation from all sources compared with 2021. To show the fluctuation in concrete terms, for example, Benin recorded the highest percentage decline of 78% while Niger and Namibia, respectively, recorded 993% and 681% increase during this period (figure 4). Similarly, in 2020 compared with 2019, 41% of countries had reductions in government spending on vaccines used in routine immunisation per surviving infant, while 31% had a reduction in 2021 compared with 2020 (figure 4).

The overall expenditure on vaccines and delivery of services and the percentage of government contribution has also witnessed a similar trend.^41^ More than 50% of domestic funding of immunisation activities reflects a positive trend towards sustainability. However, in 2022, 27 (57%) countries reported funding < 50% of their vaccine costs from domestic sources, with only 11 (23%) countries funding their vaccine cost at 50% and higher. However, this is an improvement compared with 2018 when more countries (32; 68%) were funding <50% of their expenditure on vaccines from domestic sources.^41^ Anecdotal evidence based on the authors’ field experiences and unpublished documents suggests that this inconsistent trend could be attributed to narrowing fiscal space due to unstable economic growth for various reasons, increasing debt burden and increased competition for government resources within and outside the health sector. Additionally, factors including emergencies and security challenges have further limited African governments’ ability to fund immunisation.^42^ These have a few programme repercussions such as poor and untimely forecasting and planning of immunisation activities, particularly VPD outbreak response and fractures in vaccines and cold chain supply management systems, all of which contribute to the stagnation in immunisation coverage in the region. Furthermore, the introduction of new and more expensive vaccines has added to the immunisation financing burden of African governments.

### Immunisation governance

Good progress has been made on immunisation governance and oversight in the region. Between 2010 and 2022, the percentage of African countries which established National Immunization Technical Advisory Groups (NITAGs) improved from 19% to 79%. NITAGs have contributed to better governance of immunisation programmes through the provision of evidence-informed policy and operational advice to immunisation policy makers and programme managers.^43^ This, it is believed, has contributed to better technical guidance to the programme, with resulting improvements in immunisation strategies, resources availability and coverage. However, these NITAGs operate at different levels of functionality. For instance, based on the NITAG Maturity Assessment Tool, the number of African countries with standing NITAGs increased from 38 in 2022 to 42 in 2023. However, 10 (24%) of the 42 did not meet the six basic functionality criteria of NITAGs such as availability of resources and secretariat support, integration into health policy making processes and independence (unpublished WHO/African Regional Office (AFRO) report). Additionally, financial and human resources continue to challenge the full functionality of African NITAGs. At the regional level, the Regional Technical Advisory Group (RITAG), an independent regional immunisation advisory body, continues to meet regularly and provide guidance for immunisation activities and strategies for country adaptation. It provides strategic guidance on vaccines and immunisation for regional public health organisations, such as the WHO/AFRO, including advising NITAGs on adapting regional recommendations at the country level, guiding immunisation responses to outbreaks and emergencies and identifying key areas for research and policy development.

Effective and evidence-based planning is a critical prerequisite for strong immunisation programmes. This has been enhanced by the introduction of immunisation country multiyear plans (cMYPs) which are being transitioned to the National Immunization Strategy (NIS) based on the 2022 updated guidance on developing strategic plans for immunisation. As of 2024, all 47 countries of the region have developed cMYPs and/or NIS. The key areas included in the NIS include, among others, sections and indicators on new vaccines introduction, immunisation demand, VPD control initiatives and zero-dose strategies. The development of NIS is expected to boost robust stakeholders’ engagement. However, observations show that these occur at various intensities across African countries.

## Immunisation success factors in the WHO African region

The foregoing analyses show tremendous progress in the implementation of EPI in the African region in the last five decades. Nevertheless, several challenges and unfinished agendas persist. The successes achieved so far can be attributed to five main factors.

First, the launch of the Universal Childhood Immunization Initiative by UNICEF and WHO in 1984 led to a significant increase in DPT3 coverage in developing countries by the 1990s.^44^ Additionally, the establishment of the Strategic Advisory Group of Experts (SAGE) in 1999 provided a strategic platform for delivering timely, practical and evidence-based technical guidance to African countries. SAGE has been instrumental in driving the introduction of new vaccines and technologies, further contributing to increased immunisation coverage.^8^

Second, increased political commitment due to high-level advocacy by global and regional public health organisations has placed the immunisation agenda high on the political landscape of the region. The endorsement of the Global Vaccine Action Plan, which paved the way for the decade of vaccines 2011–2020 and the launch and implementation of the Immunization Agenda 2030 (IA 2030) at the global level galvanised the much-needed political, technical and financial support to immunisation in Africa. The ADI adopted by African Heads of State in 2017^15^ was to provide the much-needed political impetus for advancing the immunisation agenda on the continent, and several African countries have laid strategies to translate the declaration into action.^45^

Third, the deployment of new innovations and digital health technologies has been critical for improving immunisation coverage globally and in Africa. The deployment of digital health technologies has improved the availability and quality of immunisation data for tracking unreached or dropout children and monitoring and improving the cold chain to ensure uninterrupted service delivery at the last mile, thereby contributing to increased immunisation coverage.^46^ Specifically, the introduction of the electronic immunisation registry for monitoring immunisation service delivery and reminder service to caregivers has contributed significantly to the good performance of the routine immunisation programmes in Tanzania,^47^ Zambia^48^ and Rwanda.^49^ Beyond these examples, the COVID-19 pandemic provided opportunities for countries to explore additional opportunities for the deployment of innovations and digital health technologies.^50^ For example, existing digital platforms such as Uruguay’s Immunization Information System and India’s Electronic Vaccine Intelligence Network (eVIN) offer valuable tools that African countries could adopt to enhance immunisation programmes management. These tools could support real-time monitoring of childhood vaccination status, data collection on vaccine safety and optimisation of vaccine deployment. Additionally, they could support vaccine logistics, including supply management, cold chain monitoring and stock control, helping to prevent stockouts.^50^

Fourth, the use of national policies that strengthen financial sustainability, health system strengthening, gender and primary healthcare approaches has contributed to solving health system and community-linked immunisation service delivery challenges in some African countries. For instance, in Rwanda, the decentralisation of immunisation services to the village and district levels and deployment of the unique local practice of ‘*imihigo’,* through which leaders sign performance contracts which are tied to specific targets, has significantly contributed to the achievement of universal immunisation coverage rates in the country.^51^ Similarly, the consistent emphasis on and funding of primary healthcare and immunisation activities in Eritrea has also resulted in impressive gains in immunisation coverage rates.^52^

Fifth, the establishment of Gavi resulted in a significant increase in the funding of immunisation activities globally and in Africa with good returns on investments.^53^ Between 2000 and 2022, Gavi partnered with 40 African countries to support vaccines against 18 infectious diseases (including COVID-19, Ebola and malaria) to the tune of US$11.9 billion which represents 58% of the body’s overall budget.^54^ Although domestic financing of vaccination remains a major challenge in many African countries, countries such as Seychelles and Mauritius are fully funding their routine immunisation programmes, which we believe contributed to the universal immunisation coverage rates achieved by these countries.

Sixth, the technical assistance provided by global and regional public health organisations, including UNICEF and WHO/AFRO, has been instrumental in strengthening African countries’ capacities for immunisation programme coordination, oversight, planning, implementation, supervision, monitoring and evaluation. For example, the framework for strategic planning for immunisation, the cMYPs developed by these agencies with active support for its rollout in countries across the AFR contributed in no small measure to programme success.^55^ Additionally, the establishment of various immunisation training programmes such as the mid-level management and vaccinology courses has increased the national capacity for immunisation programme management.

## The unfinished business: how do we address the challenges moving forward?

Moving forward, the concerted efforts of all regional and national immunisation stakeholders are required to create an enabling environment to leapfrog progress towards the attainment of the IA 2030 goals. We believe this will significantly contribute to further reductions in VPD morbidity and mortality and ultimately to the attainment of the SDGs including UHC. Finishing these unfinished businesses requires a few actions.

First, national immunisation programmes should devise innovative strategies to reach zero-dose and underimmunised children, particularly those in armed conflict-affected and hard-to-reach zones, urban slums and other underserved communities. Unvaccinated children are key drivers of VPD outbreaks. Therefore, the interest in scaling up coverage with priority to zero-dose children will have maximum impact on VPD prevention and control, address vaccine equity issues and contribute towards the SDGs. Increasing investments in community-based health initiatives, mobile medical teams and the deployment of digital health technologies and strategies such as RED and the GPEI’s house-to-house immunisation campaign will provide the platform to expand routine immunisation services to such marginalised populations.^56 57^

Second, African countries should continue to strengthen primary healthcare and promote resilient health systems as platforms for improving immunisation service delivery in line with the IA 2030. Specifically, immunisation services should be integrated into other high-quality Primary Health Care (PHC) services like child and adolescent health, newborn and women health, nutrition, etc. Furthermore, health system strengthening interventions should be used as opportunities to address the systemic challenges that hinder the effective delivery of immunisation and other health services across the life course. In this regard, the immunisation for the PHC framework for action has identified strategic levers around political commitment, governance, funding and engagement of stakeholders.^58^ In the area of governance, the Interagency Coordinating Committee, which exists at the national level and has been used to effectively coordinate partners around the polio eradication initiative and COVID-19, should be deployed to strengthen coordination of routine immunisation.

Third, the challenge of vaccine hesitancy due to sociocultural reasons, which has remained a major barrier to routine immunisation demand, should be comprehensively addressed using context-specific and evidence-based strategies. We recommend the use of sociological and anthropological evidence to understand the factors that drive vaccine hesitancy at national, district and village levels and the planning of evidence-based communication and community engagement and participation interventions to address them.^59^

Fourth, African countries should expand tax-based revenue to increase government distributable revenue, continue to protect immunisation budgets and remove budget execution bottlenecks to increase efficiency and ensure timely vaccine procurement and deployment. Additionally, immunisation services should be included as a part of the benefit package of national health insurance schemes as a way of increasing domestic funding^60 61^ while also scaling up high-level advocacy to political leaders to increase domestic funding of immunisation.^62^ Countries with thriving private and business sectors should tap into resources that might be available from collaboration with the private sector. In line with the Lusaka Agenda on donor resource alignment and the Accra Agenda on harmonisation and alignment, we recommend better prioritisation of immunisation resources and funding.^63 64^ This approach would enhance efficiency gains, allowing for reinvestment in scaling up immunisation services. However, partner funding will be essential to quickly ramp up coverage in the short term, as efforts to increase domestic resources are being made, taking into cognisance the call to countries to increase domestic funding as contained in the ADI.^15^

Fifth, VPD outbreaks which result in avoidable morbidity, mortality and disruption of immunisation services need to be effectively addressed. Prioritising timely and effective outbreak response activities and rapidly addressing low immunisation coverage are therefore essential. Countries should enhance outbreak preparedness planning, especially in low coverage areas, by ensuring prepositioning of vaccines, cold chain, immunisation supplies and funding for timely outbreak response. Addressing the root cause of outbreaks, low routine immunisation coverage, requires catch-up campaigns and accelerated routine immunisation efforts.

Sixth, African countries should scale up implementation research to identify gaps in service delivery and vaccine demand uptake, document successes and innovative strategies and identify, implement and evaluate locally tailored immunisation solutions. Additionally, the regional public health organisations should support countries to scale up basic research and development aimed at adapting new technologies and innovations to support EPI implementation and identify more effective vaccine delivery strategies, particularly in hard-to-reach areas and slums. Furthermore, countries should harness digital technological solutions to improve the timeliness, completeness and quality of disaggregated routine immunisation and immunisation financing data to improve evidence-based planning for service delivery.

Seventh, increasing the supply of routine immunisation vaccines through localisation of vaccine production in the region is imperative moving forward. This requires increased political commitment, capacity building, adequate funding and research and development. Existing organisations and partnerships, such as the Partnership for African Vaccine Manufacturing, the African Vaccine Regulatory Forum and the African Medicine Agency, should be supported to effectively fulfil their regulatory mandates.^40^ African governments should use the Gavi-supported African Vaccine Manufacturing Accelerator Platform which is availing US$1 billion to African countries to promote local vaccine manufacturing. Additionally, efforts to ensure equitable access to vaccines should be strengthened within the Africa Continental Free Trade Area Agreement framework.

Lastly, regional public health organisations, such as WHO/AFRO, UNICEF and the Africa Centres for Disease Control and Prevention, should provide the necessary technical assistance to support countries in implementing these initiatives. This includes offering guidance on immunisation policies and strategies while continuously monitoring and reporting on regional immunisation coverage trends and other key indicators. Additionally, through RITAG, regional partners should facilitate high-level advocacy efforts to scale up and secure sustainable financing for immunisation programmes, as well as support capacity building and research.

## Conclusion

Immunisation plays a crucial role in addressing global health challenges and development goals. This underscored the establishment of EPI in 1974 as a vehicle to rapidly increase routine immunisation coverage globally. Five decades into its implementation in Africa, the programme has made significant progress, but huge challenges and several unfinished businesses persist. The 50th anniversary of the programme calls for renewed commitments of African governments to ensure every African child is immunised, thereby laying the foundation for achieving the SDGs including UHC. Efforts to increase immunisation coverage in the African region should focus on accelerating the implementation of the IA 2030 and the ADI commitments, addressing the COVID-19 setbacks, leveraging new technologies and securing sustainable immunisation funding. The lessons from the implementation of the GPEI and the COVID-19 pandemic response would also be valuable in this regard. Additionally, scaling up implementation research to identify gaps in service delivery and vaccine demand uptake and to implement and evaluate locally tailored immunisation solutions will contribute to improving immunisation service coverage in the region. We call on all African immunisation stakeholders, including African political and public health leaders, the international vaccine industry, regional and international public health organisations, the academia, donors and healthcare workers to support countries in the realisation of this noble objective.
